# A New Method of Castrating Horses

**Published:** 1899-09

**Authors:** 


### SURGICAL ITEMS.

*A New Method of Castrating Horses.* Dr. Hoffmann, professor of surgery at the Royal Veterinary High-School in Stüttgart, through the columns of the Berlin *Veterinary Weekly*, advocates the following technique for performing aseptic castrations:

To limit the struggling as much as possible, without administering a general anæsthetic, the horse is given a full dose of chloral one hour before operating. He is secured in the usual position, and the operating region thoroughly scrubbed with soap and water, and then bathed with a solution of lysol. After drying the part moderately with sterilized cotton, thioform is applied, by first dusting it over the surface and then rubbing it into the skin until an even coating is formed over the entire scrotum and immediate vicinity. The testicle is then exposed in the usual manner, but is pulled from the scrotum with a pair of iron tongs instead of with the hand. The cord is then clasped with a second pair of clamp-like tongs, which are furnished with a screw to tighten and retain the hold. The testicle is then removed by simple torsion with the first pair of tongs. Before as well as after letting loose with the second pair the cavity is poured full of the following solution: thioform, 30 gm.; sodium bicarbonate, 10 gm.; glycerin, 60 gm. After removing the second testicle in the same manner, the whole field is anointed with an ointment consisting of thioform, 5 parts, and lanolin, 50 parts. The thioform and glycerin mixture is said to adhere firmly to the exposed tissues and perfectly prevent infection during the healing process.

Prof. Hoffmann makes mention of the fact that the hands of the operator cannot be kept disinfected while castrating a horse, hence the necessity of adopting a method whereby the hands will not come in contact with the wound.

The method is well worth a trial. Thioform is a comparatively new dry antiseptic, and is quoted in Merck's catalogue at seventy-five cents an ounce.

If the method is adopted in this country I predict that the technique will be improved, as the American practitioner will be very slow in discarding the now popular écraseur and emasculator for the torsion recommended above. The introduction of either of these instruments instead of the iron clamp-like tongs will certainly prove an improvement, and at the same time either can be used without touching the wound with the hands, which is the chief object of the method.—L. A. M.

